# Influence of age, BMI and parity on the success rate of midurethral slings for stress urinary incontinence

**DOI:** 10.1371/journal.pone.0201167

**Published:** 2018-08-16

**Authors:** Rosa Maria Laterza, Ksenia Halpern, Daniela Ulrich, Alexandra Graf, Karl Tamussino, Wolfgang Umek, for the Austrian TVT vs. TVT-O Study Group

**Affiliations:** 1 Department of Obstetrics and Gynaecology, Division of General Gynaecology and Gynaecologic Oncology, Medical University of Vienna, Vienna, Austria; 2 Karl-Landsteiner-Institut fuer Spezielle Gynaekologie und Geburtshilfe, Vienna, Austria; 3 Department of Obstetrics and Gynaecology, Medical University of Graz, Graz, Austria; 4 Center for Medical Statistics, Informatics and Intelligent Systems, Medical University of Vienna, Vienna, Austria; University Medical Center Utrecht, NETHERLANDS

## Abstract

**Aims:**

Our aim was to evaluate, in a second data analysis of the prospective randomized controlled trial conducted by Austrian Urogynaecology Working Group, the effect of age, BMI and parity at the time of surgery on short- and long-term outcomes of women primarily treated for SUI (stress urinary incontinence) with midurethral slings.

**Methods:**

In the original study 554 patients received randomly a retropubic (TVT) or a transobturator midurethral (TVT-O) sling procedure. 480 (87%) and 277 (50%) patients were available for a follow-up efficacy evaluation at 3 months and 5 years respectively.

**Results:**

Higher age and BMI at surgery appear to lead to a larger probability to have a positive stress test 5 years after surgery, but not after 3 months. Older patients seem to have a worse perception of improvement 5 years after surgery as compared to younger ones, as described by the PGI-I score. Age and BMI do not affect significantly the quality of life of women surgically treated for SUI, as reflected by the results of King´s Health Questionnaire. Parity does not seem to have any effect on objective and subjective surgical outcomes.

**Conclusions:**

Higher age and BMI at surgery have a detrimental influence on the objective cure rate at 5 years after midurethral sling surgery; higher age also has a negative influence on subjective long-term outcomes. However, these demographic parameters do not influence significantly the quality of life of patients after anti-incontinence surgery. Parity does not show any significant influence on success rate of midurethral sling.

## Introduction

Retropubic tension-free vaginal tape (TVT) or transobturator tape (TOT) are a standard of care for the surgical treatment of stress urinary incontinence in women [1]. These two procedures have been shown to have comparable outcomes with subjective cure rates of up to 85% [1–3].

Several studies have demonstrated the feasibility and safety of midurethral slings in elderly [4–8] and obese [9–12] women.

However, the literature shows significant variability in terms of influence of demographic characteristics on midurethral sling outcomes [11–13].

The Austrian Urogynaecological Working Group conducted a randomized controlled trial (RCT) to compare objective and subjective outcomes of TVT with those of TVT-O [3,14]. The aim of this study was to perform a secondary analysis of these data, in order to assess if age, BMI and parity at the time of surgery may influence the short- and long-term outcomes of women primarily treated for SUI with midurethral tapes.

## Materials and methods

The current study is a secondary analysis utilizing an established database from a previously reported prospective randomized controlled noninferiority study by the Austrian Urogynaecology Working Group [3,14]. The aim of this study was to compare objective and subjective outcomes of TVT versus TVT-O as primary treatment for female stress urinary incontinence (SUI). The Ethics Committee ofthe Medical University of Graz and the institutional review boards at Wilhelminenspital Wien; Univ.-Frauenklinik Wien; Univ.-Frauenklinik Innsbruck; LKH Mödling; LKH Leoben; Krankenhaus der Barmherzigen Brüder Graz; LKH Klagenfurt; Krankenhaus der Barmherzigen Brüder Wien; LKH Wiener Neustadt; Krankenhaus der Barmherzigen Schwestern Linz; Krankenhaus Amstetten; LKH Bad Ischl; LKH Judenburg; LKH Dornbirn; LKH Wels; LKH Feldbach; Krankenhaus der Barmherzigen Brüder St. Veit; LKH Gmunden; BKH Schwaz; Donauspital Wien; KH Korneuburg; Hanusch KH; Amper-Klinikum Dachau; Klinik für Frauenheilkunde/Campus Innenstadt, University of Munich approved the original study protocol. It was planned according to Consolidated Standards of Reporting Trials (CONSORT) guidelines [15] and registered with ClinicalTrials.gov (NCT 00441454). Women were eligible for inclusion in the RCT when they had an urodynamically verified SUI (positive cough stress test at bladder filling of 300 ml) without concomitant prolapse surgery or hysterectomy and when they were suitable candidates for midurethral tape procedures. Women were excluded from participation if they had detrusor overactivity or a predominant complaint of overactive bladder, concomitant prolapse surgery, other major concomitant surgery (i.e., hysterectomy), previous incontinence surgery other than colporraphy, residual urine ≥ 100 ml, neurologic diseases, allergy to local anesthetic agents, coagulations disorders or other contraindications for surgery. Women were randomized to receive TVT or TVT-O procedure, according to the description of the original study [3], where detailed description of the study methods was presented.

For the RCT, preoperative evaluation included a demographic questionnaire (including age, height, weight and parity), urogynaecological history, bladder diary, urodynamic studies (cystometry and urethral profilometry) and cough stress test. Condition-specific quality of life (QoL) was assessed with the validated German-language version of the King’s Health Questionnaire (KHQ) [16], and with the validated German-language version of Patient Global Impression of Severity (PGI-S) and Patient Global Impression of Improvement (PGI-I)[17].

Postoperatively, participants were evaluated at 3 months and at 5 years: both evaluation included a clinical and urodynamic evaluation, with a cough stress test at bladder filling of 300 ml; patients were also asked to complete the same QoL questionnaires used for the preoperative evaluation.

Cough stress test was performed in the supine and standing positions at bladder filling of 300 ml. Patients were considered objectively cured when this test in the supine and standing positions were negative.

Secondary analysis of the RCT data, evaluated if age, BMI and parity affect the objective and subjective cure rate of SUI at 3 months and 5 years postoperatively. Objective measurement of SUI cure was considered as negative cough stress test at 3 months and at 5 years postoperatively. Subjective cure was measured at 3 months and 5 years using QoL questionnaires (KHQ, PGI-S and PGI-I). King’s Health Questionnaire were assessed in 3 parts according to Hebbar et al. [18]: Part I assesses general health perception and incontinence impact; Part II evaluates role limitations, physical limitations, social limitations, personal relationship, emotions, sleep/energy, severity measures; Part III is considered as a single item and contains ten responses in relation to frequency, nocturia, urgency, urge incontinence, stress incontinence, nocturnal enuresis, intercourse incontinence, infections, bladder pain and postvoid dribble.

For this analysis we assumed that TVT and TVT-O have comparable success rate, as demonstrated by our original study [3] and by others trials [1,2]. The BMI was calculated using the standard formula of kg/m^2^.

The STROBE Statement guidelines for reporting observational cohort research were followed [19].

### Statistical analysis

To analyse the influence of age, BMI and parity (as continuous variable) on the probability of a positive stress test, logistic regression models (one for 3 month and one for 5 year results) were performed. To analyze the results in more detail, polynomial regression models (using the LOESS approach) were performed for the influence of age and BMI. To investigate the influence of parity in more detail, proportions of failing stress test and corresponding 95%-confidence intervals were calculated for each observed parity value. The three parts of the King’s Health Questionnaire were calculated according to Hebbar et al. [18]. The influence of age, BMI and parity on the scores were analysed using linear regression models (separately for 3 months and 5 years results). Additionally, Pearson correlation coefficients were calculated. The difference in age, BMI and parity between PGI-S and PGI-I classes were analysed using ANOVA. Due to the small number of patients with PGI-I score >4, the PGI-I was grouped in 4 classes scores (Classes 1, 2, 3 and >4). To get an overview of the data, histograms and scatter plots were plotted. For descriptive description of the data means, standard deviations (SD), as well as median, 25% (Q1) and 75% (Q3) quantiles, Minimum (Min) and Maximum (Max) were calculated. To verify if all underlying assumptions of each statistical testing method were satisfied, we analysed the residual plots and the histograms. All analyses were performed using R Software, release 3.2.1. All p-values smaller than 0.05 were considered as statistically significant.

## Results

**Fig 1:**
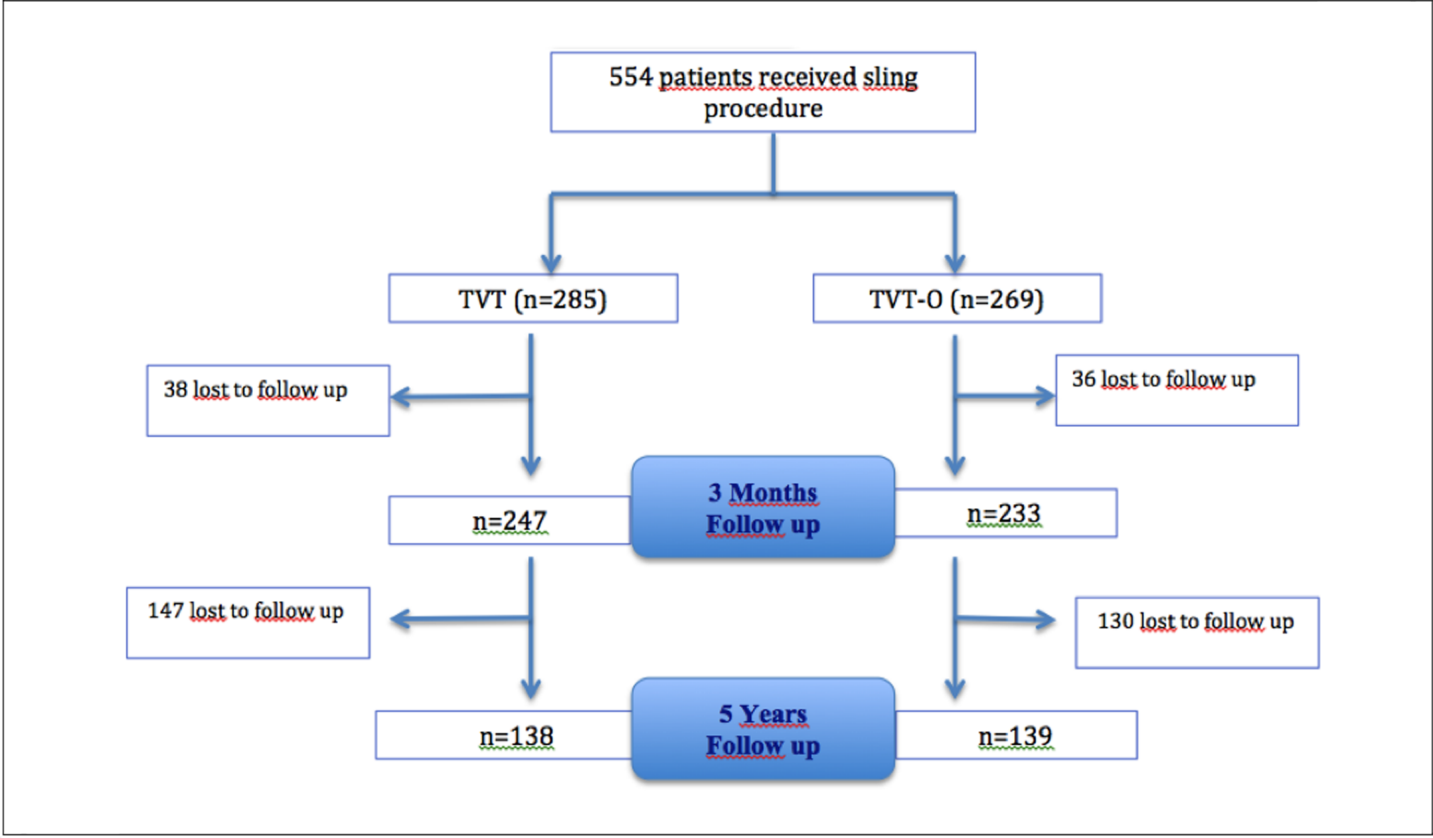
Consolidated Standards of Reporting Trials (CONSORT) guidelines. Flow Chart.

A total of 554 patients were included in this study between January 2005 and July 2007: 285 (51%) TVT and 269 (49%) TVT-O. Of them, 480 (87%) patients were assessed at 3 months and 277 (50%) patients at 5 years. (Fig 1)

At the time of surgery, mean age was 59.2 (SD± 11.0; min 33-max 85) years, mean BMI was 28.1 (SD±5.11; min 15-max 62), median parity was 2 (range 0–8). The intraoperative complications occurred were: 11 (1.9%) bladder perforations, 5 (0.9%) intraoperative bleeding. Reoperations due to postoperative complications were 5 (0.9%).

**Fig 2:**
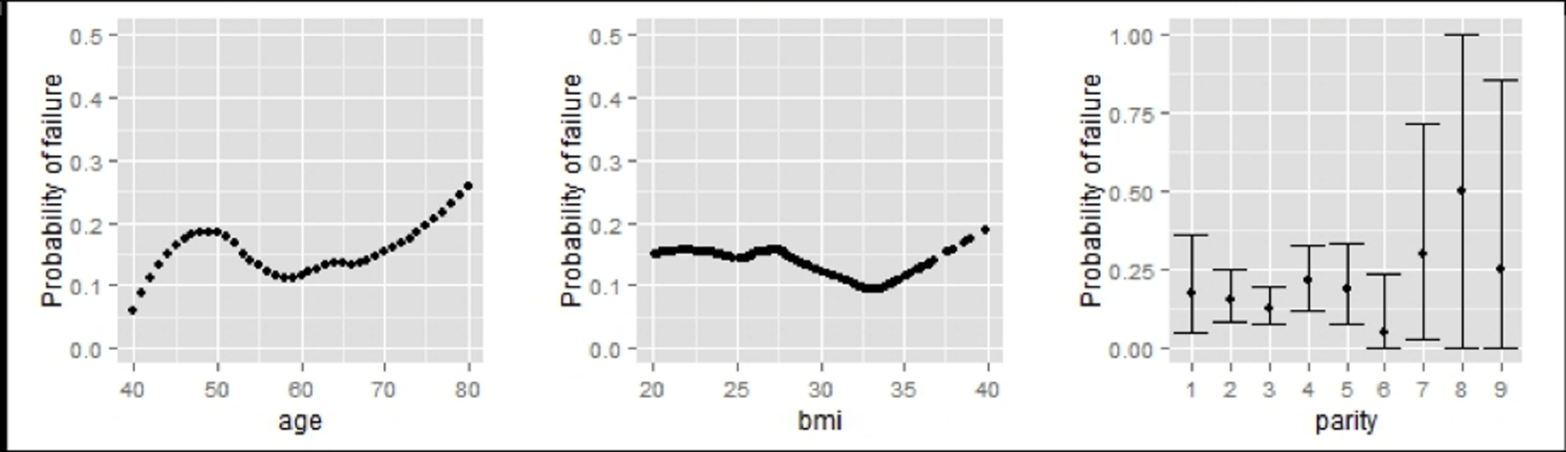
Probability to have a positive stress test (probability of failure) at 3 months depending on age, BMI and parity.

At 3 months a positive stress test was seen in 70/480 (16%) women. At 3 months no significant influence of age, BMI and parity on a positive stress test was found (p = 0.23, p = 0.52 and p = 0.91 respectively). However, the polynomial regression model showed a trend for an increasing probability for surgery to fail with increasing of age. The probability of stress test failure seems to be rather constant for all BMI values. Also for parity, no discernible pattern was identified (Fig 2).

**Fig 3:**
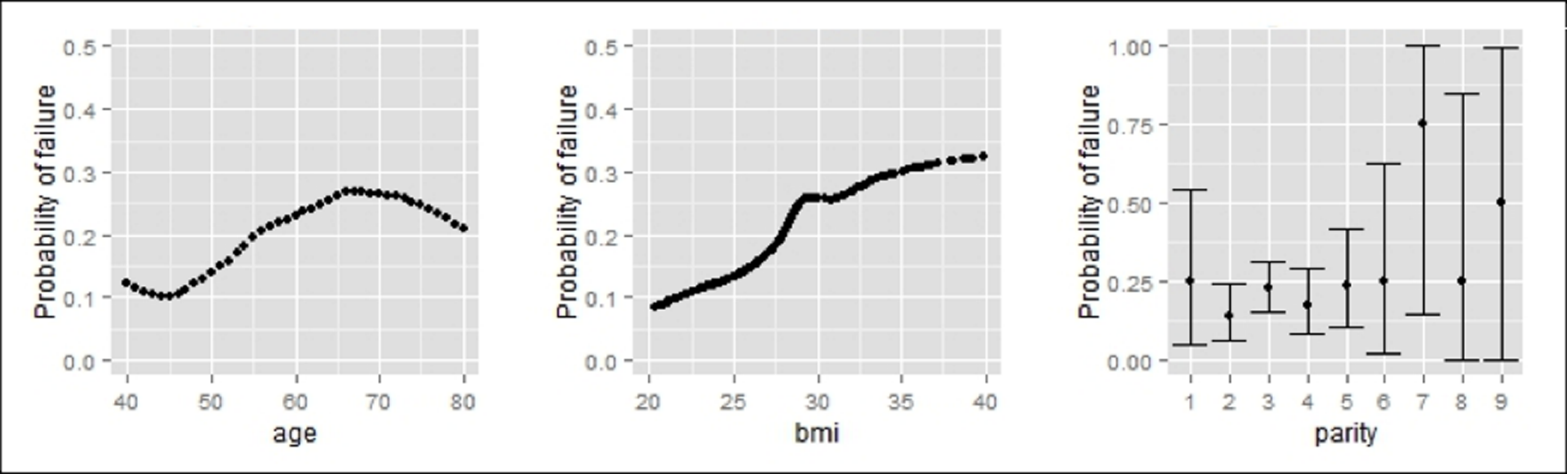
Probability to have a positive stress test (probability of failure) at 5 years depending on age, BMI and parity.

At 5 years a positive cough stress test was seen in 56/277 (20%) women. The probability to have a positive stress test at 5 years was significantly increasing with age (p = 0.05) and with BMI (p = 0.01). This trend can also be seen using polynomial regression models (Fig 2). For parity, also after 5 years, no discernible pattern was found, as reflected in the result of the logistic regression model (p = 0.50) (Fig 3).

A significant difference in mean BMI between PGI-S classes at 3 months after surgery was observed: patients with lower PGI-S scores (those with better perception of urinary tract condition) had higher BMI values. A reverse trend, although not significant, was found after 5 years. No significant influence of age and parity on PGI-S scores at 3 months and 5 years was found (Table 1).

**Table 1:** Descriptive statistics (mean ± standard deviation) and results of ANOVA models for age, BMI and parity compared between PGI-S score classes at 3 months and 5 years.

		PGI-S Classes				
		1	2	3	4	p-value
3 Months	N	235	66	19	13	
	Age	60.10 ± 10.63	57.94 ± 11.62	63.86 ± 12.76	60.78 ± 9.61	0.30
	BMI	28.43 ± 4.48	27.49 ± 5.41	28.65 ± 4.19	24.11 ± 3.14	**0.05**
	Parity	2.08 ± 1.26	2.34 ± 1.34	2.38 ± 1.04	2.38 ± 1.77	0.18
5 Years	N	149	61	34	14	
	Age	58.66 ± 10.44	59.23 ± 12.22	62.39 ± 12.05	64.57 ± 10.61	0.11
	BMI	27.73 ± 5.64	28.21 ± 4.59	28.03 ± 4.50	30.76 ± 4.76	0.29
	Parity	2.19 ± 1.14	2.05 ± 1.18	2.10 ± 1.14	2.36 ± 2.01	0.90

At 3 months a significant difference of BMI between the PGI-I classes was found. On average a higher BMI was found for patients with lower PGI-I scores (those with a better perception of improvement as compared with before treatment). However, at 5 years, although not significant, a reverse trend was found. A significant difference in age was found between PGI-I classes after 5 years: older patients had higher PGI-I classes (worse perception of improvement as compared with before treatment). No significant difference in parity between the PGI-S classes was found, neither 3 months nor 5 years after surgery (Table 2).

**Table 2:** Descriptive statistics (mean ± standard deviation) and results of ANOVA models for age, BMI and parity on PGI-I score classes at 3 months and 5 years.

		PGI-I Classes				
		1	2	3	≥ 4	p-value
3 Months	N	221	72	27	14	
	Age	59.40 ± 10.96	59.61 ± 9.80	63.45 ± 13.68	60.00 ± 9.60	0.27
	BMI	28.65 ± 4.49	27.45 ± 5.45	27.14 ± 3.80	26.45 ± 4.63	**0.03**
	Parity	2.17 ± 1.33	2.14 ± 1.15	1.81 ± 0.98	2.44 ± 1.01	0.71
5 Years	N	157	48	24	36	
	Age	57.72 ± 10.52	59.54 ± 12.03	62.08 ± 10.50	65.60 ± 9.83	**<0.0001**
	BMI	27.64 ± 5.43	28.06 ± 4.67	29.04 ± 5.06	28.76 ± 4.79	0.16
	Parity	2.16 ± 1.07	2.37 ± 1.33	2.05 ± 1.15	1.97 ± 1.51	0.47

The descriptive statistics of the three scores of KHQ are described on Table 3.

**Table 3:** Descriptive statistics of the distribution of the three scores of the KHQ for 3 months (first row) and 5 years (second row). Lower scores indicate patient wellbeing and higher scores mean that the person is severely affected by urinary incontinence.

King’s Health Questionnaire									
		Mean	Std	Min	Q1	Median	Q3	Max	N
3 Months	Part1	48.79	41.93	0	25	33	75	180	350
	Part2	113.53	134.35	0	33	67	130	620	200
	Part3	23.78	5.44	6	20	25	28	30	271
5 Years	Part1	55.88	42.55	0	25	50	83	180	255
	Part2	146.76	131.28	0	61	99	190	690	230
	Part3	23.93	5.39	3	21	25	28	30	227

Age, BMI and parity did not significantly influence any of the three KHQ scores at 3 months and 5 years (all p-values >0.05) (Table 4).

**Table 4:** Regression coefficients of the regression model describing effect of age, BMI and parity on three parts of KHQ as well as the corresponding Pearson correlation coefficient.

King’s Health Questionnaire						
		Variable	Estimate	Standard Error	p-Value	Correlation Coefficient
3 Months	Part1	age	0.00	0.25	0.99	-0.01
		BMI	-0.88	0.59	0.14	-0.10
		parity	-0.68	2.18	0.76	-0.02
	Part2	age	0.20	1.02	0.84	0.00
		BMI	-4.08	2.32	0.08	-0.15
		parity	-5.17	9.34	0.58	-0.07
	Part3	age	-0.04	0.04	0.31	-0.06
		BMI	0.14	0.08	0.07	0.13
		parity	-0.01	0.31	0.99	0.00
5 Years	Part2	age	0.36	0.25	0.15	0.11
		BMI	-0.12	0.51	0.82	-0.02
		parity	2.56	2.32	0.27	0.07
	Part2	age	0.79	0.81	0.33	0.08
		BMI	-0.60	1.64	0.71	-0.03
		parity	0.24	7.74	0.86	0.01
	Part3	age	-0.03	0.03	0.42	-0.06
		BMI	0.04	0.07	0.57	0.03
		parity	-0.06	0.32	0.86	-0.01

## Discussion

This secondary analysis of data from a large prospective randomized trial of the surgical outcomes of tension-free vaginal tape shows that at 3 months, the patient´s age or BMI did not have a significant influence on either objective and subjective outcomes. While at 5 years older patients with higher BMI had a higher probability of a positive stress test. Parity had no significant influence on surgical outcomes of midurethral slings.

The results of our study indicate that age and BMI at surgery may affect the objective long-term outcomes of SUI surgery, but not the early. This is plausible considering that the immediate mechanical effect of midurethral slings is independent of ageing of the tissue and patient´s weight. When placed correctly, a midurethral sling supports the urethra in a “hammock-like” way and shows its curative effect after 3 months. However, after 5 years, the remodelling process around the tape and the deterioration of pelvic floor support occur. During this time the overweight may affect pelvic floor structures thereby, increasing intra-abdominal pressure in older and overweight patients, resulting in the loss of the therapeutic effect of the sling. In fact, the ageing of pelvic floor tissue is due to decreased number of vascular plexuses and collagen type II/III content in the urethral submucosa and to changes in striated pelvic floor muscle.This is a risk factor for the deterioration of the continence mechanisms, and consequently, also for the efficacy of anti-incontinence surgery [13]. Further, the prolonged increased intra-abdominal pressure of overweight women has an additional detrimental effect on this process.

Controversial results were found with PGI-S and PGI-I questionnaires which indicated that patients with higher BMI defined their urinary tract condition as normal and had a better global impression of improvement 3 months after surgery than patients with lower BMI. However at 5 years a reverse trend was found. This can be explained by the immediate mechanical effect of midurethral sling at 3 months after surgery in the more overweight patients, which perceived a greater impression of improvement than in thinner women. This effect is lost over the time, particularly in the more overweight patients, since the prolonged increased intra-abdominal pressure counteracts the urethral support. This interpretation should however take in account that from 3 months to 5 years, several patients were lost to follow up. These results could therefore be also a result of selection bias.

At 5 years, there is a trend showing that older and overweight women perceive a severe urinary tract condition and a diminished perception of improvement. However this was only significant for age. This shows that the long-term subjective outcomes seem to be influenced by age and BMI also.

On the other hand, our results show that age, BMI and parity at the time of surgery do not significantly affect the QoL of patients after anti-incontinence surgery. This can be seen in all the KHQ´s domains: distress, anxiety, loss of self-esteem, social, cultural, marital, domestic, physical, psychological and sexual wellbeing related to urinary incontinence, are not significantly influenced by age, weight and parity at the time of surgery.

There is little consensus in literature about the influence of age and weight on surgical success of patients candidates for suburethral slings. That can be explained by the use of different outcome parameters and different follow up timing utilized in the different trials.

Our results, studying long-term follow-up are consistent with those reported by Anger et al [20], who demonstrated that treatment failure 12 months postoperatively was higher in patients over 75 years than in patients between 65 and 75 years old (10.5% vs 7.2%). Similarly, Rechberger et al [13] demonstrated that menopausal status and ageing negatively influenced the surgical outcomes at 18 months. In contrast, Stav et al [6], in a multivariate analysis of more than 1200 midurethral tapes, recently showed that age was not a risk factor for surgical failure at 24 months.

Regarding BMI, some authors have shown that BMI did not influence the midurethral sling success rates [13,21,22] within 27 months of follow-up. In contrast Hellberg et al [23] found a decrease of subjective cure rates at mean follow-up of 5.7 years in the obese patients, which is in line with our findings. No randomized controlled trials related to the topic are to our knowledge available in literature.

A recent review [24] of 2846 patients, including 6 prospective cohort studies [13,21,25–28] and 5 retrospective studies [11,29–32], concluded that the objective success rates of midurethral sling are lower in overweight and obese patients. However, the subjective outcomes were not significantly different among normal weight, overweight and obese patients. The authors suggested that surgeons should not consider BMI>25 kg/m^2^ as a risk factor when discussing the suitability of the midurethral sling procedure in a patient with SUI. Some important limitations have to be taken in account when assessing this meta-analysis. First, the follow-up times are different among the different studies, ranging from 6 months [21,28] to 86.4 months [25]. Therefore, the results might have been different with different follow-up times. Moreover, there was no standard method for assessing the outcomes of surgery.

Our finding that higher BMI at surgery leads to a larger probability to have a positive stress test 5 years after surgery but not 3 months after surgery are supported by the fact that all the outcome parameters were standardized, validated and uniformly applied at 3 months and 5 years postoperatively.

The strength of this analysis is that it was performed in one of the largest prospective randomized trial of retropubic vs. transobturator midurethral tapes. The post hoc statistical power calculation with the present sample size is >90%, with an a error of 0.05. The study population was homogeneous and representative, including peri/post-menopausal (mean age 59± 11 years) and overweight (mean BMI 28.1± 5) women at the time of surgery. Additionally, the study had clear, robust and validated outcome measures.

One limitation of the study is the fact that 50% of patients were lost to 5 years follow-up. All attempts to reach study-patients for follow-up within the scope of the ethics-committee agreement were made such as contacting by telephone, mail and e-mail. Attempts we also made by inquiring new residential address from the national registration office, arranging at least three possible follow-up appointments as well as sending postal questionnaires to women who did not attend the clinical appointment. As previously described [14], 58% of our patients responded to questionnaires at 5 years, but only 50% were available to complete the clinical examination. Therefore, the reasons for missing data within this trial are that the patient were unreachable by telephone, mail or e-mail, patients withdrawal as well as patients not available to reach the hospital for the clinical examination. No withdrawal of participating centres from the trial occurred.

However, to date there have been few randomized trials of surgery for stress incontinence with long-term follow up; it has to be taken in account that it is one of the few studies reporting on 277 patients prospectively 5 year after midurethral sling procedures.

## Conclusions

Higher age and BMI at surgery have a detrimental influence on the objective and subjective long-term outcomes of midurethral tape surgery. On the other hand, our results suggest that these demographic parameters do not affect the QoL of patients after anti-incontinence surgery. As population demographics continue to evolve, specifics on age-related and weight-related outcomes of urinary incontinence interventions deserve further investigations. The results of this study add robustness and validated evidence on risk factors of treatment failure of midurethral sling procedures, giving the clinician the ability to provide correct and exhaustive information, that better modulates a patient's expectations.

## Supporting information

S1 File Data used for the statistical analysis. Data used for the statistical analysis of the study, collected in excel files.
(ZIP)
